# Super-Enhancers and CTCF in Early Embryonic Cell Fate Decisions

**DOI:** 10.3389/fcell.2021.653669

**Published:** 2021-03-25

**Authors:** Puja Agrawal, Sridhar Rao

**Affiliations:** ^1^Department of Cell Biology, Neurobiology, and Anatomy, Medical College of Wisconsin, Milwaukee, WI, United States; ^2^Versiti Blood Research Institute, Milwaukee, WI, United States; ^3^Department of Pediatrics, Medical College of Wisconsin, Milwaukee, WI, United States

**Keywords:** CTCF, enhancer, nanog, pluripotency, embryonic stem cell

## Abstract

Cell fate decisions are the backbone of many developmental and disease processes. In early mammalian development, precise gene expression changes underly the rapid division of a single cell that leads to the embryo and are critically dependent on autonomous cell changes in gene expression. To understand how these lineage specifications events are mediated, scientists have had to look past protein coding genes to the *cis* regulatory elements (CREs), including enhancers and insulators, that modulate gene expression. One class of enhancers, termed super-enhancers, is highly active and cell-type specific, implying their critical role in modulating cell-type specific gene expression. Deletion or mutations within these CREs adversely affect gene expression and development and can cause disease. In this mini-review we discuss recent studies describing the potential roles of two CREs, enhancers and binding sites for CTCF, in early mammalian development.

## Introduction

Understanding the molecular underpinnings of cell fate decisions is a central question within developmental biology. For decades it has been assumed that these decisions are mediated almost exclusively by the 2% of DNA that contains protein-coding genes, while the remaining 98% was considered “junk.” Over time, scientists have realized that this 2% of DNA could not exclusively explain all the developmental and disease-related decisions made during early mammalian development. This has given rise to a renewed focus on the remaining 98% of the genome, including non-coding DNA sequences and nuclear architecture and their role(s) in driving cell fate decisions by regulating gene expression. Nuclear architecture is the dynamic, three-dimensional organization of chromatin and its interaction with itself and regulatory proteins within the nucleus. The development of high-resolution imaging and chromosome conformation capture technologies, which permit a direct interrogation of long-range DNA:DNA interactions, has facilitated these important aspects, which regulate gene expression. Two types of *cis-*regulatory elements (CREs), which play a critical role in modulating gene expression, are enhancers and DNA-binding sites for CTCFs. In this mini-review we will focus on both classes of CREs specifically, as well as their mechanisms and roles in driving early cell fate decisions during early mammalian development. The examples discussed below do not encompass all the literature available by any measure but reflect selected key studies in the field focusing on embryonic stem cells (ESCs). For further information on enhancer and CTCF functions, we suggest these reviews (Ong and Corces, 2014; Shlyueva et al., 2014; Li et al., 2016; Chen et al., 2018; Rowley and Corces, 2018; Agrawal et al., 2019; Braccioli and De Wit, 2019).

## Early Mammalian Development

Early placental mammalian development is characterized by numerous rapid, distinct cell fate decisions (Arnold and Robertson, 2009). Upon fertilization, an embryo is totipotent: it can form any cell type, both embryonic and extraembryonic tissues. After a few cell divisions the cells become pluripotent: they can form the three germ layers (mesoderm, endoderm, and ectoderm) but not extraembryonic tissue. At the blastocyst stage two distinct populations are formed: trophectoderm and the pluripotent inner cell mass (ICM). The trophectoderm, which eventually gives rise to the placenta, expresses *CDX2*, which turns off the pluripotency gene *Pou5f1*, which encodes the pluripotency critical transcription factor Oct3/4 (Strumpf et al., 2005; Rayon et al., 2016). The ICM maintains pluripotency by continuing expression of Oct3/4, which subsequently differentiates into the epiblast (EPI) and the primitive endoderm (PrE). The EPI eventually gives rise to all fetal tissues and maintains pluripotency by expressing *Nanog*, while the PrE, which will form the yolk sac, differentiates by down-regulating *Nanog* and expressing *Gata6* (Messerschmidt and Kemler, 2010; Schrode et al., 2014). Development of the embryo is thus dependent on the proper temporal and spatial gene expression changes to drive lineage commitment. Experimentally, embryonic stem cells (ESCs) are derived from the early embryo and exist in multiple states, naïve (ICM) and primed (epiblast; Ghimire et al., 2018). These states are distinct with respect to transcription, where primed cells show a relative down-regulation of pluripotency genes and an up-regulation of lineage-specific genes, compared with the naïve embryos, with additional differences, indicating that pluripotency is not a single-cell state.

## Enhancers

Enhancers are CREs that contain DNA-binding motifs for transcription factors (TFs) distal to the gene promoter, which are located immediately adjacent to the transcriptional start site (TSS). Binding of these TFs drives enhancers to promote or repress gene expression through multiple mechanisms that are beyond the scope of this review (Li et al., 2016; Chen et al., 2018; Agrawal et al., 2019). Next-generation sequencing-based (NGS) methods have revolutionized the genome-wide identification of enhancers. The combination of chromatin immunoprecipitation (ChIP) with NGS (ChIP-Seq) initially permitted the identification of potential enhancer regions based on p300 and H3K4me1 enrichment (Visel et al., 2009; Rada-Iglesias et al., 2011). These regions can be further subdivided based upon the enrichment of the mutually exclusive marks H3K27me3 and H3K27Ac, indicating whether an enhancer is “poised” or “active,” respectively. Genome-wide study of histone and TF enrichment has led to the identification of a distinct class of enhancers termed super-enhancers. Super-enhancers (SEs, Whyte et al., 2013) are a highly active minority of enhancers defined by their high density of binding lineage-specific TFs and associated cofactors such as the Mediator complex and p300. SEs also exhibit relatively higher levels of H3K27Ac and are larger in size overall compared with enhancers (Parker et al., 2013; Pulakanti et al., 2013). Several studies show that SE differ from classical enhancers due to their stronger ability to drive gene expression than classical enhancers (Hnisz et al., 2015; Huang et al., 2016; Shin et al., 2016; Thomas et al., 2021). SEs are also highly cell-type specific and are often found near key lineage determining genes, implying they are critical to the establishment and/or maintenance of cell identity. For example, in ESCs there are 231 super-enhancers (Whyte et al., 2013), which are defined by their enrichment for the pluripotency TFs Nanog, Oct4, and Sox2, as well as Mediator and high levels of H3K27Ac, and many are in close proximity to pluripotency-promoting genes, including *Nanog.*

**FIGURE 1:**
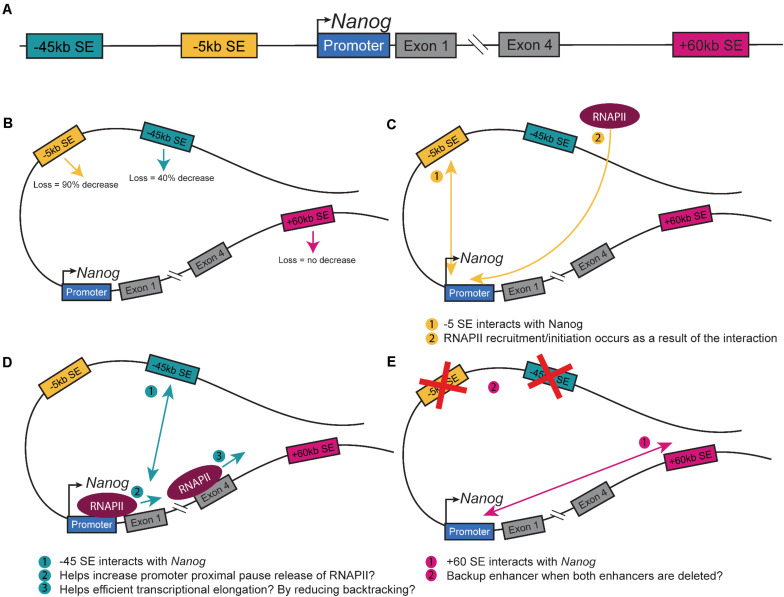
Nanog Super-enhancers. **(A)** Schematic of the *Nanog* extended locus with the three SEs. Schematic of a theoretical loop containing all three SEs and Nanog depicting. **(B)** The changes in *Nanog* expression upon deletion (Blinka et al., 2016; Agrawal et al., 2021). **(C)** The interaction between the −5SE and *Nanog* and how it alters RNAPII dynamics (Agrawal et al., 2021). **(D)** The interaction between the −45SE and *Nanog* and its potential mechanisms. **(E)** The interaction between the +60SE and *Nanog* and the possible backup role it has in regulating the gene.

The master pluripotency TF *Nanog* is near three SEs (−5SE, −45SE, +60SE, named based on distance from *Nanog* promoter in kb; Figure 1A; Pulakanti et al., 2013; Whyte et al., 2013; Blinka et al., 2016; Agrawal et al., 2021). These SEs and the numerous CTCF sites at the locus make it an ideal model system to understand how CREs regulate cell fate decisions by modulating *Nanog* expression (Blinka and Rao, 2017). Mouse-derived embryonic stem cells (mESCs) are an ideal model for studying enhancer-promoter interactions, which promote *Nanog* expression. Due to their pluripotent nature, they are sensitive to changes to gene expression with a simple observation of cell fate, differentiation. Tracking *eGFP*-tagged *Nanog* and isolating GFP^–^ cells revealed that cells not expressing *Nanog* could give rise to *Nanog* expressing cells and continue to self-renew (Chambers et al., 2007). These cells do, however, give rise to fewer undifferentiated colonies, indicating that down-regulating *Nanog* predisposes but does not proscribe cells to differentiation. At the single-cell level, *Nanog-*low cells begin to down-regulate pluripotency-associated genes and up-regulate lineage-specific gene markers, while maintaining pluripotency (Abranches et al., 2014). Blastocyst division into the EPI and PrE is dependent on lineage specific gene expression and the location of the cells (Xenopoulos et al., 2015). One of the first markers of this division of cell fates is *Nanog*, wherein the presence of *Nanog* denotes EPI cell fate, while the loss of *Nanog* marks the development of PrE. As the ICM differentiates, there are a few PrE to EPI conversions, but no EPI to PrE conversions, indicating that the EPI state is a distinct form of pluripotency from the ICM. In culture, *Nanog* is expressed highly in naïve pluripotent ESCs but fluctuates in primed ESCs (Barral et al., 2019). Thus, the modulation of *Nanog* is important to understand, as the delicate regulation of the gene’s expression is critical to defining pluripotent states. Circular Chromosome Capture (3C) of the three SEs at the *Nanog* locus demonstrates that they physically interact with the *Nanog* promoter, but CRISPR-based deletion of each SE differentially alters expression (Blinka et al., 2016). Deletion of the −45SE causes an approximately 50% decrease in *Nanog* expression, deletion of the −5SE causes a nearly 90% decrease, but deletion of the +60SE causes no change (Figure 1B; Blinka et al., 2016; Agrawal et al., 2021). This leads to the important question: through what mechanism(s) do the −5SE and −45SE regulate *Nanog*, expression? Interestingly, single-cell RT-qPCR showed that deleting the −5SE altered *Nanog* expression in all cells demonstrates that it is functional in all cells. Additionally, analysis of RNA Polymerase II (RNAPII) dynamics upon deletion of the −5SE shows a complete loss of RNAPII at the *Nanog* promoter, indicating that the −5SE modulates *Nanog* expression by recruiting RNAPII or by promoting its conversion to its initiating form through phosphorylation of Ser 5 of the C-terminal domain (CTD, Figure 1C). Further analysis of the −45SE and its role in regulating *Nanog* gene expression is necessary to determine whether the two enhancers utilize the same or distinct mechanism(s) from the −5SE (Figure 1D; Henriques et al., 2018; Chen et al., 2018; Bartman et al., 2019; Sheridan et al., 2019; Zatreanu et al., 2019; Noe Gonzalez et al., 2020).

Other lineage-critical genes are also regulated by enhancers. The expression of *Shh*, a gene critical at multiple stages of development in many tissues, is critically regulated by enhancers (Anderson and Hill, 2014). In the zone of polarizing activity (ZPA) in the developing limb, mutations in the ZPA regulatory sequence (ZRS) cause polydactyly due to misregulation of *Shh* in early limb development (Lettice et al., 2003). The ZRS is interesting as it is one megabase away from *Shh*, displaying just how distant the regulation of some genes can be. The interaction of the ZRS and *Shh* is specific to E10.5–11.5 mouse embryos, limiting the ZRS activity to limb development stages (Williamson et al., 2016). Interestingly, the ZRS, a highly conserved regulatory region in vertebrates, consists of multiple discrete enhancer elements that regulate gene expression and long-range interactions, emphasizing the multiple and varied roles of enhancers in regulating development (Lettice et al., 2017). In the brain, numerous Shh-Brain-Enhancers (SBEs) modulate the spatiotemporal expression of *Shh* in the developing midbrain (Jeong et al., 2006; Yao et al., 2016). Curiously, deletion of the SBEs can alter *Shh* expression in neural progenitor cells and brain development (Jeong et al., 2008; Benabdallah et al., 2016), but 3D-FISH analysis of *Shh* and SBEs shows an increase in distance between the gene promoter and regulatory element, implying a mechanism independent of looping (Benabdallah et al., 2019).

*Sox2*, another pluripotency gene, has multiple enhancer regions, some of which have no effect on pluripotency when deleted but have a potential role in neural cells (Ferri et al., 2004). Other regions, specifically the *Sox2* Control Regions (SCR) disrupt maintenance of pluripotency in ESCs when deleted (Zhou et al., 2014). Similarly, to *Shh*, although the SCR is critical to *Sox2* transcription, recent live cell imaging shows that in ESCs, the SCR and *Sox2* are not in close physical proximity within the nucleus, indicating they operate at a distance (Alexander et al., 2019).

As an example of the interface between development and disease, enhancers play a critical role in the neural-crest-derived craniofacial disorder, the Pierre Robin sequence (PRS), which is classically characterized by mandibular underdevelopment along with other characteristics (Long et al., 2020). PRS is linked to mutations in a Topologically Associated Domain (TAD, described below), containing a single protein coding gene, *Sox9* (Gordon et al., 2014). *Sox9* plays a critical role during neural crest differentiation and craniofacial development (Lee and Saint-Jeannet, 2011; Dash and Trainor, 2020; Nagakura et al., 2020) and enhancers near *Sox9* have been implicated in craniofacial and chondrocyte development (Liu and Lefebvre, 2015; Yao et al., 2015). PRS associated mutations are >1Mb from *Sox9* and near a cluster of three enhancers the deletion of which leads to a 50% allele-specific decrease in *Sox9* expression (Long et al., 2020). These three clusters are enriched for activating chromatin marks exclusively in the developing human neural crest cells (hNCC), indicating a cell-type specific role for these enhancers. In a mouse model, deletion of one of these enhancer clusters causes a modest 13% decrease in *Sox9* expression that is sufficient to cause mandibular developmental changes and adversely effect survival, indicating that a dose-dependent change in *Sox9* expression due to enhancer cluster deletion alters mandibular development. Enhancer control of the *Shh, Sox2* and *Sox9* loci demonstrate how CRE modulation of gene expression allows one gene to play multiple roles at various points in development.

Although all the nuances of how enhancers determine cell fate are still being investigated, we do understand that their role is critical to a range of developmental processes. Two mechanistic insights not discussed here but also under investigation are whether and how enhancers modulate gene expression by regulating transcriptional bursting (Bartman et al., 2016, 2019; Fukaya et al., 2016) and/or phase separation (Cho et al., 2018; Sabari et al., 2018; Zamudio et al., 2019). Analysis of *Shh* and *Sox2* enhancers has shown that physical proximity may not be necessary, further expanding the question of the mechanisms that enhancers use to modulate gene expression.

## CTCF

**FIGURE 2:**
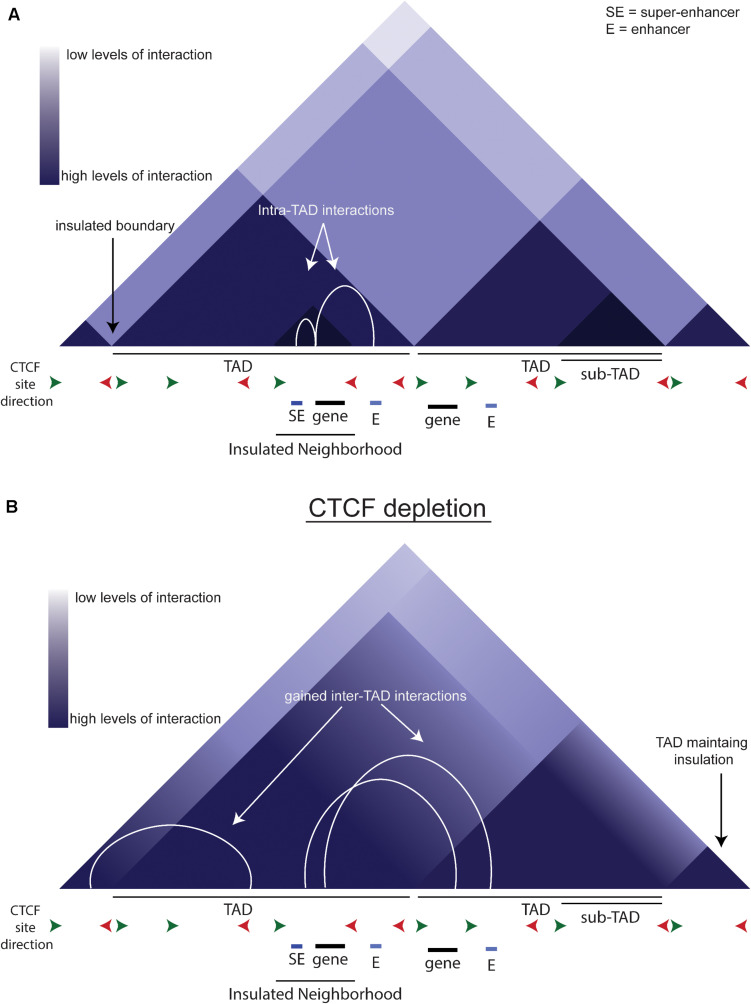
TADs and CTCF. **(A)** Schematic of chromatin organization. The triangles depict a schematic of a Hi-C contact map. White arcs show the intra-TAD interactions that are insulated by CTCF. **(B)** Schematic of what a Hi-C contact map may look like upon CTCF depletion. White arcs show the gained interactions across previous TAD boundaries.

DNA is organized within the nucleus on multiple levels (Ong and Corces, 2014; Rowley and Corces, 2018). Double-helix DNA is packaged into chromatin, which is further folded into nucleosomes that can be regulated by histone modifications. The development of 3C-based techniques has allowed the study of chromatin-chromatin interactions across large genomic distances (Dekker et al., 2002; de Laat and Dekker, 2012). Hi-C, a technique that studies long range chromatin-chromatin interactions, has led to the definition of Topologically Associated Domains (Figure 2A, TADs). TADs are large regions of chromatin, bounded by CCCCTC binding factor (CTCF) binding sites, in which the majority of interactions remain within the TAD. CTCF is a DNA-binding insulator protein and has binding sites present throughout the genome, within and at the boundaries of TADs. TADs can be further broken down to sub-TADs which are cell-type specific insulated regions within a TAD bounded by CTCF and cohesin (Figure 2A, Phillips-Cremins et al., 2013; Dowen et al., 2014; Hnisz et al., 2016). These sub-TADs are found to contain tissue or development-specific genes. Insulated neighborhoods (INs) are among the types of sub-TAD specifically identified in ESCs described as a CTCF-CTCF loop containing an SE and its target gene (Dowen et al., 2014). Deletion of one boundary CTCF site at key pluripotency genes (i.e., *Nanog, Pouf51*) is sufficient to alter gene expression, indicating these INs facilitate proper gene expression regulation, perhaps by creating and/or preventing enhancer-promoter interactions. A key point to consider is that INs and TADs were identified using different techniques and therefore may be identifying the same phenomenon but at different scales due to methodological differences.

The insulator protein CTCF contains 11 highly-conserved zinc-finger DNA binding domains (Phillips and Corces, 2009; Ong and Corces, 2014; Rowley and Corces, 2018). Insulator proteins have numerous functions, including regulating intrachromosomal interactions and the spread of heterochromatin. Knockout of CTCF is embryonic lethal at E5.5, and depletion of maternal CTCF in oocytes adversely impacts blastocyst stage embryos (Moore et al., 2012). CTCF has pleiotropic effects on gene expression, including altering DNA methylation and affecting splicing, as well as the regulation of enhancer-promoter interactions through chromatin looping (Ong and Corces, 2014). Current dogma supports an extrusion model of chromatin looping between two CTCF-bound sites that are in convergent orientation in collaboration with the cohesin complex (Rao et al., 2014; de Wit et al., 2015). The cohesin complex is a ring structure made up of four protein components, Rad21, Smc1, Smc3, and Stag1/2 (Fisher et al., 2017). While its primary function is to stabilize sister chromatids during cell division, cohesin also plays a critical role in maintaining chromatin loops, often in conjunction with CTCF. But 50–80% of CTCF binding sites are co-localized with cohesin. Although depletion of cohesin does not alter CTCF occupancy, there is a loss of looped domains, indicating that CTCF-dependent looping relies on cohesin (Rao et al., 2017). Further analysis of the CTCF protein revealed that mutating the N-terminus reduces TAD insulation (Nora et al., 2019). The orientation of the CTCF binding site positions the N-terminus to interact with cohesin, providing a molecular explanation of the necessity of convergent CTCF sites to create a chromatin loop. The necessity of cohesin and CTCF binding for chromatin looping, however, is currently being questioned, as two recent students have shown that depletion of CTCF and cohesin does not affect a majority of enhancer:promoter interactions (Thiecke et al., 2020; Kubo et al., 2021).

Many studies have shown that CTCF plays a role during development (reviewed in Arzate-Mejía et al., 2018). At the *HoxA* locus, deletion of CTCF sites within the locus alters the enrichment of the repressive H3K27me3 mark, permitting aberrant *HoxA* gene expression during the differentiation of ESCs to motor neurons (Narendra et al., 2015). In the developing heart, deletion of the CTCF gene in cardiac progenitor cells causes embryonic lethality at E12.5, through loss of chromatin interactions and, potentially, enhancer:promoter interactions at key developmental genes (Gomez-Velazquez et al., 2017). As a distinct example, inhibiting CTCF function prevents Schwann cell differentiation and causes hypomyelination *in vivo* (Wang et al., 2020). These are a few examples of the variety of roles CTCF plays in gene expression regulation, but little is known on how to identify what role CTCF is playing modulating nearby genes and whether there are biological signs that differentiate one CTCF site’s function from another.

In an auxin-inducible depletion system in mESCs, loss of CTCF reduces proliferation, indicating that CTCF is integral to the maintenance of pluripotency (Nora et al., 2017). The acute depletion of CTCF causes an overall loss of looping anchored by CTCF and cohesin (Figure 2B). There is also an increase in inter-TAD interactions that is reversed upon auxin wash-off, implying that CTCF normally prevents these interactions. Interestingly, while CTCF depletion causes loss of insulation on most boundaries, some boundaries remain intact (Figure 2B), indicating that the role of CTCF is not universal across the genome. CTCF sites are more likely to be near genes up-regulated by CTCF depletion, and these genes are more likely to be near active enhancers, so they are normally separated by a TAD boundary. Thus, the loss of the TAD boundary likely permits aberrant enhancer-promoter interaction(s) causing the up-regulation of these genes. In conclusion, CTCF is critical to the precise regulation of gene expression by modulating enhancer:promoter contacts.

## Super-Enhancers and CTCF

Several studies have focused on the interaction between CTCF and enhancers, specifically SEs. CTCF has been found to be bound near most SEs and within some SEs (Dowen et al., 2014; Ing-Simmons et al., 2015; Huang et al., 2017). One study finds that a mammary gland specific SE modulates not only the mammary gland specific gene *Wap*, but also the non-mammary-specific *Ramp3. Ramp3* lies outside of the chromatin loop formed by two CTCF sites flanking *Wap* and the associated SE. Deletion of the separating CTCF sites greatly increases the interaction between the SE and *Ramp3* and *Ramp3* expression, thus showing that the CTCF sites insulate against this interaction to maintain proper expression (Willi et al., 2017). In a variety of cancers, SEs are gained near *MYC* to up-regulate *MYC* and drive oncogenic transformation. These SEs lay between two CTCF sites that form an IN neighborhood and modulate expression (Schuijers et al., 2018). These studies, and others (Hay et al., 2016; Hanssen et al., 2017; Shin, 2019), support the theory that CTCF is critical to SE-mediated gene expression regulation.

## Discussion

The full potential of CTCF sites and enhancers in regulating gene expression is still being investigated and a number of questions remain. As an example, in view of the evidence that CTCF sites modulate SE function, at the *Nanog* locus, what is CTCF’s role? Is the IN truly the two sites just 5′ and 3′ of the gene, as implied in Dowen et al., and can other nearby CTCF sites compensate? And are these CTCF sites encouraging and/or preventing enhancer:promoter interactions? Beyond *Nanog*, the question still remains whether there are different classes of CTCF binding sites that can be identified through a biological signature. Given that loss of CTCF can cause an up-regulation and down-regulation of gene expression, how can we identify which role it plays for any given gene? Similarly, there are still questions regarding enhancers and their role in cell fate decisions: specifically are all super-enhancers and the same? It is clear from the data regarding the *Nanog* +60E that this cannot be true, because although it has all the defining features of a super-enhancer, in ESCs, at least, it does not play a significant role. Perhaps it is a redundant enhancer, becoming fully functional if the −5SE and −45SE are out of commission or is utilized in other cell types such as primordial germ cells (Figure 1E). Can we identify different features of SEs and assign them to different categories? The field of nuclear architecture remains open to investigation, and as we further study chromatin-chromatin interactions, the minute control of gene expression control will clarify how cell fate decisions are made and how this process breaks down in disease pathology.

## Author Contributions

PA wrote the first draft of the manuscript. SR and PA edited the manuscript together. Both authors contributed to the article and approved the submitted version.

## Conflict of Interest

The authors declare that the research was conducted in the absence of any commercial or financial relationships that could be construed as a potential conflict of interest.
